# National trends in pediatric drowning — insights from the Israeli Ministry of Health registry-based cohort

**DOI:** 10.1007/s00431-024-05771-5

**Published:** 2024-09-16

**Authors:** Yael Applbaum, Malena Cohen-Cymberknoh, Adi Avniel-Aran, Ayala Yahav, Ezra Weinblatt, Rebecca Brooks, Joel Reiter, Shulamit Gordon, Ziona Haklai, Uri Pollak

**Affiliations:** 1 Division of Health Information, Ministry of Health, Jerusalem, Israel; 2 Pediatric Pulmonary Unit and CF Center, Hadassah University Medical Center, Jerusalem, Israel; 3 Faculty of Medicine, The Hebrew University of Jerusalem, Jerusalem, Israel; 4 Section of Pediatric Critical Care, Hadassah University Medical Center, 9112001 Jerusalem, Israel; 5 Department of Pediatrics, Hadassah University Medical Center, Jerusalem, Israel

**Keywords:** Pediatric drowning, Ministry of Health, Deaths

## Abstract

This study analyzes the epidemiology of pediatric drowning in Israel from 2010 to 2022, focusing on differences across age groups, sex, and regions. We conducted a retrospective cohort study using data from the Ministry of Health’s administrative databases, covering all children aged 0–17 years who were seen in the emergency department, discharged after hospitalization, or died at the scene due to drowning, excluding cases of intentional harm or suicide. The primary outcome was the annual drowning rate per 100,000 children, categorized by age, sex, and region, along with hospitalization duration, intensive care unit admissions, and mortality rates. A total of 2101 drowning incidents were identified, with 9% resulting in death. Higher drowning rates were found in younger children (1–4 years) and teenagers (15–17 years), with notable differences by sex. Drowning rates were higher in coastal regions and more frequent during winter and spring. An increase in drowning rates was observed over the last 3 years of the study (2020–2022). Children hospitalized for more than 3 days had significantly higher mortality rates, both in general wards and intensive care units.

*Conclusions*: Pediatric drowning is a persistent public health concern in Israel, with distinct seasonal, regional, age-specific, and sex-specific patterns in incidence and mortality. This study underscores the need for a comprehensive prevention strategy that includes year-round public education, environmental safety measures, and targeted interventions for high-risk groups to reduce drowning incidents and fatalities among children effectively.

**Table:** 

**What is Known:** *• Pediatric drowning represents a significant public health challenge globally, with varying rates.* *• In Israel, pediatric drowning is the second leading cause of death from all injury-related deaths. Thus far, the measures and interventions to reduce fatalities were not proven effective enough.*	
**What is New:** *• Pediatric drowning in Israel, with a 9% mortality rate, revealed a biphasic rate varied by sex and is higher in coastal regions and during winter and spring.* *• There was a notable increase in drowning incidents during the last 3 years of the study period (2020–2022), coinciding with the COVID-19 pandemic.*

**Supplementary Information:**

The online version contains supplementary material available at 10.1007/s00431-024-05771-5.

## Introduction

Drowning is defined as experiencing respiratory impairment due to submersion or immersion in liquid and can result in both fatal and nonfatal outcomes [1]. It often occurs due to unsafe water-related behavior, inadequate supervision, and environmental factors in settings like homes, swimming pools, natural water bodies, and recreational areas. Drowning is a major public health concern and a leading cause of death among children and youth globally [2]. The death rate varies widely, from 3 per 100,000 among children aged 1 to 4 in the USA [3] to 107 per 100,000 among 2 years old in Thailand [4]. In Australia, drowning rates are 200 times higher than traffic deaths when adjusted for exposure [5]. In Israel, drowning is the second leading cause of accidental death among children, accounting for 16–18% of cases [6].

Drowning incidents also impose significant economic burdens, with costs estimated at over US $273 million annually in the USA [7], over US $228 million in Brazil [8], and US $1.24 billion AUD in Australia [9]. Despite decreasing rates of childhood drowning in the USA, it remains the leading cause of unintentional injury-related death in children aged 1–4 years, surpassing birth defects as of 2018 [10]. Toddlers are particularly at risk due to their mobility and curiosity [11, 12], while teenagers face increased risks from decreased supervision and risk-taking behaviors [13], as well as factors like trauma and substance use [14]. Health conditions such as long QT syndrome [15], epilepsy [16], and autism spectrum disorder [17, 18] are also linked to increased drowning risk.

While Israel has implemented regulations and safety measures, such as lifeguard requirements at public pools and beaches, challenges persist, especially regarding unregulated private pools and disparities in access to swimming education [19]. There is a lack of comprehensive, long-term studies on pediatric drowning, limiting understanding of trends and risk factors. This study aims to analyze the epidemiology of pediatric drowning in Israel from 2010 to 2022, exploring differences by age, sex, season, and region to inform a national prevention strategy.

## Methods

### Study population and data collection

This retrospective cohort study examined accidental drowning among children aged 0–17 years in Israel from 2010 to 2022. Data were collected from the Ministry of Health’s administrative databases, including the National Hospital Emergency Department Registry (NHEDR), the National Hospital Discharges Registry (NHDR), and the National Cause of Death Registry (NCDR). These databases provided encrypted patient information, demographics, diagnoses, and outcomes, allowing data matching through uniform encryption.

Children with accidental drowning diagnoses were included, excluding intentional harm or suicide cases. Drownings were identified using ICD-9 codes in the NHEDR and NHDR and ICD-10 in the NCDR. Institutional review board approval was obtained before data collection.

**Fig. 1:**
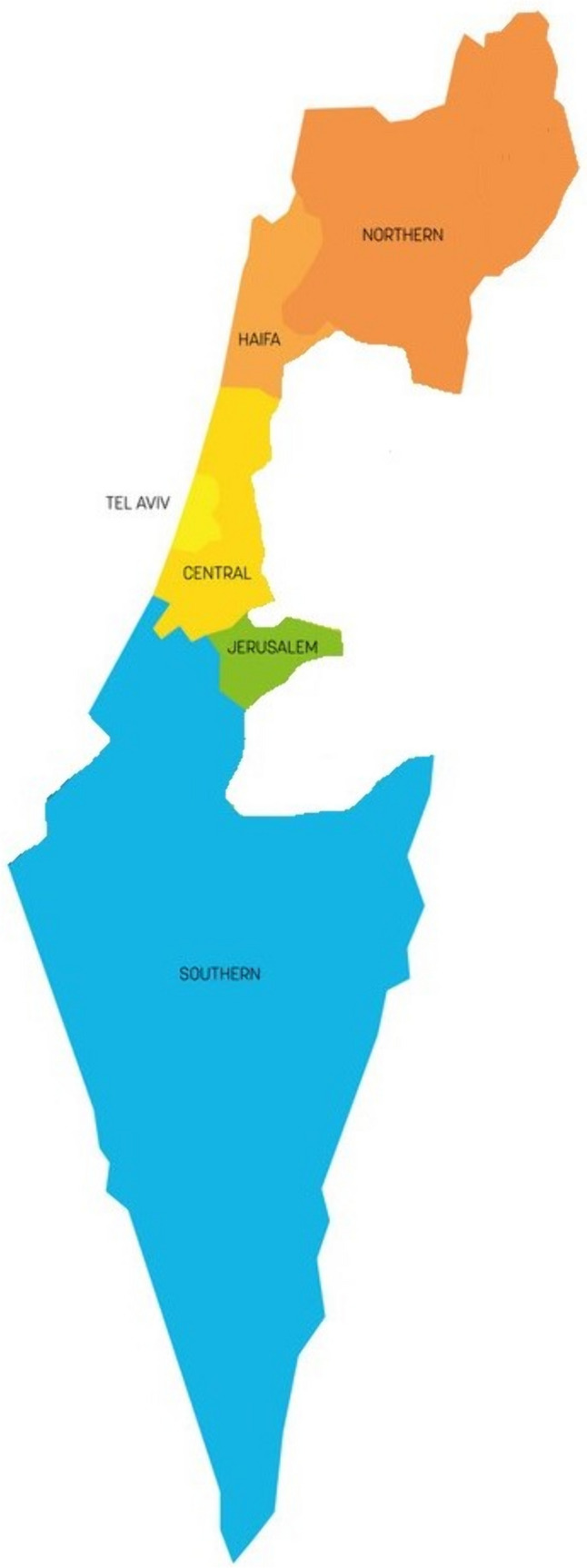
The map of Israel by districts. Israel is divided into six districts according to differences in geography and the major metropolitan areas. The Mediterranean Sea is on the right-hand (West) side of the country

Data were analyzed by age, sex, residence, season, year, and outcome. Drowning severity was assessed based on hospitalization days, ICU days, and deaths. Population data were stratified by age and adjusted for age-specific drowning rates per 100,000 children. Israel’s geographic divisions (Fig. 1) and seasonal definitions (winter (December–February), spring (March–May), summer (June–August), and autumn (September–November)) were used for analysis.

The annual pediatric population size was stratified by age during the study period using datasets available from the Central Bureau of Statistics (CBS). We described the number of accidental drownings among children aged 0–17 years and calculated age-adjusted rates per 100,000 children, stratified by age, sex, and residence.

### Statistical analysis

The primary outcome of this study was the annual drowning rate among children aged 0–17 years, stratified by age group, sex, residence, season, and outcome, and reported per 100,000 population over the 13-year study period. All variables were treated as categorical, despite being recorded as integers. Categorical variables are presented as percentages with 95% confidence intervals, unless otherwise specified. Differences between groups for these categorical variables were analyzed using either the Fisher exact test or the Pearson chi-square test, depending on the data's characteristics.

Direct age adjustment was performed to compare drowning rates by sex, residence district, and year. Specifically, the Fisher exact test was used for sex comparisons, the Pearson chi-square test for residence district comparisons, and the Cochran-Armitage trend test for year-over-year trends. All statistical analyses were conducted using SAS version 9.4. Confidence intervals were calculated using binomial proportions, and a p-value of less than 0.05 was considered statistically significant. For time trend analyses, p-values were derived from the Cochran-Armitage trend test, while comparisons between sexes used the Fisher exact test, and comparisons between regions and seasons employed the Pearson chi-square test.

## Results

**Fig. 2:**
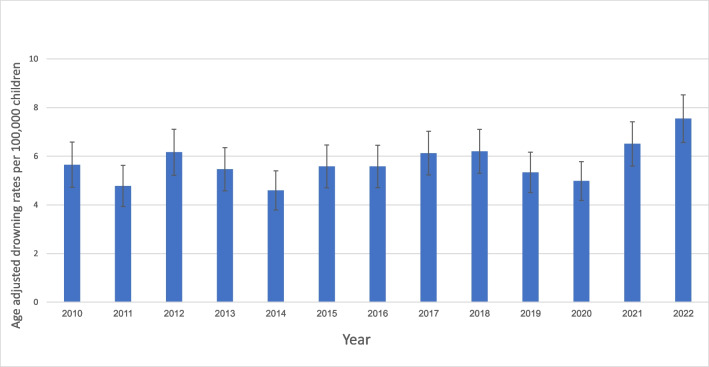
Age-adjusted drowning rates per 100,000 children aged 0–17 by year 2010–2022. Annual drowning rate during the study period. Drowning incidence was highest in 2021–2022

The study identified 2101 drowning cases between 2010 and 2022, with an increase to the highest rate in the study period in 2022 (supp. Table 1). Of those, 189 (9%) died, in 38/189 (20%) cases death was pronounced at the scene of drowning, in 64/189 (34%) in the ED, and in 87/189 (46%) during their hospitalization. There was no distinct trend in mortality over the study period. The annual rate of hospital admissions from the ED ranged between 52 and 68% with no clear trend. Drowning incidence was highest in 2021–2022, at 6.6–7.5 per 100,000 compared with 5–6 per 100,000 until 2020 (Fig. 2, supp. Table 2).

### Seasonal variations

Our analysis of the seasonal variation revealed higher rates of drowning during the summer. Conversely, a higher percentage of deaths were observed during the winter and spring, compared with the summer and autumn seasons (13% vs. 7–8%, *p* < 0.001).

### Length of hospitalization and mortality

A total of 2063 children were transferred to hospitals, of whom 57% were admitted, and the remainder were discharged home from the ED. Of children admitted to the ED, 3.1% were pronounced dead in the ED and 4.2% during their hospitalization. Hospitalization data showed that children hospitalized for 4 days or more had a notably higher mortality rate (26%) compared to those hospitalized for 1–3 days (4%). Similar trends were observed in ICU stays, where children hospitalized for 4 days, or more, had a significantly higher mortality rate compared to those hospitalized in the ICU for 1–3 days (39% vs 9%) (Table 1).

**Table 1:** Death rates of hospitalized children, both in the ward or in the ICU by duration of stay

		Drowned	Died	% died	95% *CI*	*p*-value
Hospital days§	All	1182	87	7%	(5.9%, 9.0%)	
	1–3	984	35	4%	(2.5%, 4.9%)	< 0.001
	4 +	198	52	26%	(20.3%, 33.0%)	< 0.001
ICU days§	All	461	80	17%	(14.0%, 21.1%)	
	1–3	329	29	9%	(6.0%, 12.4%)	< 0.001
	4 +	132	51	39%	(30.3%, 47.5%)	< 0.001

^§^Exact Fisher test

### Age- and sex-specific variations

**Fig. 3:**
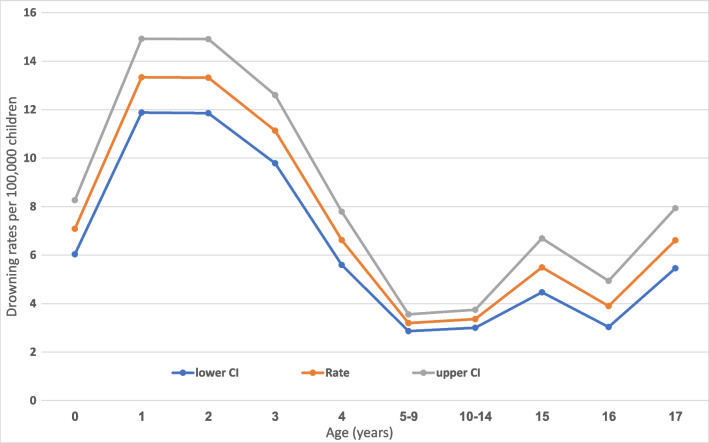
Rates of drowning among children aged 0–17 per 100,000 by age, 2010–2022. The age-specific rate is bimodal with a peak between the ages 1–4 and a lower peak between ages 15–17

A bimodal age-specific rate of drowning was identified, peaking between 1–4 and 15–17 years old (Fig. 3, supp. Table 3). The age-adjusted incidence rate for drowning during the entire study period was 5.7 (95% *CI* 5.5–6.0) per 100,000 children 0–17 years old.

There was a higher drowning rate in males (7.1 per 100,000, 95% *CI* 6.7–7.5) compared with females (4.3 per 100,000, 95% *CI* 4.0–4.6). Furthermore, the overall death rate was 10% in males compared with 7% in females (supp. Table 1). More boys died at the scene of the drowning than girls (27 vs 11), more died in the ED (47 vs 17), and more died during the hospitalization (62 vs 25).

### Regional contribution

Drowning rates varied significantly by residence region. Regional analysis showed lower rates in Jerusalem (4.6 per 100,000, 95% *CI* 4.0–5.2) and higher rates in the Northern District (6.6 per 100,000, 95% *CI* 5.9–7.2).

## Discussion

In this nationwide cohort study, we analyzed over 2000 drowning cases involving children under 17 years of age across 13 years, with 9% resulting in death. The study revealed significant variations in drowning rates by age, sex, and region. This is the first study in Israel to examine pediatric drowning over such an extended period, including all cases regardless of mortality, providing a comprehensive epidemiological overview.

The 9% mortality rate highlights the ongoing public health challenge of pediatric drowning, reflecting global trends of persistent drowning risks among children [20]. The recent increase in drowning rates observed in our study indicates an urgent need for improved preventive strategies, as current measures may have reached a plateau in effectiveness [21]. Our findings offer crucial insights for developing targeted prevention strategies and may serve as a model for similar studies and interventions worldwide.

Our study found that drowning incidents peaked in the summer, likely due to increased water-related activities, consistent with findings from Banihani et al. [22] and Loux et al. [23], who reported higher drowning rates in the United States and Florida during warm months. However, mortality rates were higher in colder seasons, such as winter and spring, possibly due to more hazardous conditions and reduced lifeguard presence. To reduce drowning risks throughout the year, recommendations include public education on water safety, improved lifeguard training, environmental modifications, community programs, and technological interventions like AI surveillance and wearable detection devices [24, 25].

The most severe drowning incidents often result in immediate death at the scene, while those who reach the emergency room (ER) show that severity, as indicated by longer hospital or ICU stays, correlates with higher mortality rates. Our study found that children hospitalized for 3 days or less had a 7% mortality rate, whereas those hospitalized for 4 or more days had a 39% mortality rate, consistent with other studies suggesting that prolonged stays indicate more severe conditions [26]. These findings underscore the importance of rapid emergency responses, including effective CPR by caregivers and bystanders [27, 28] and highlight the role of advanced inhospital resuscitation techniques, such as extracorporeal life support (ECLS), in improving survival rates, as evidenced by data from the National Trauma Data Bank and the Extracorporeal Life Support Organization [30, 31].

Our study identified a bimodal distribution in age-specific drowning rates, with peaks in children aged 1–4 and a smaller peak in teenagers aged 15–17. This pattern, along with the male predominance observed in nearly 60% of drowning cases reported by Benihani et al. [22] and higher hospitalization rates for males found by the Center for Injury Research and Policy [32], reflects broader trends linked to risk-taking behaviors and substance use [33–37]. To address these disparities, we recommend targeted education programs, enhanced supervision, and promoting safety measures such as life jackets and drowning detection devices. Ongoing research is essential to evaluate these interventions’ effectiveness.

Despite the high risk of drowning among adolescents, our study and findings by Peden et al. reveal a significant lack of targeted prevention strategies for this group, with no studies evaluating effective interventions [38]. This gap is concerning given adolescents’ unique risk factors, such as increased risk-taking behaviors, peer influence, and limited supervision. To address this, comprehensive, evidence-based strategies are needed, including education on specific risks, safety measures in recreational areas, and community-based interventions involving adolescents and their families. Filling this gap is crucial for reducing drowning incidents and enhancing prevention efforts.

Our study reveals regional disparities in drowning incidences, likely influenced by cultural practices, geographic proximity to water bodies, and access to swimming education [39]. Drowning was less frequent in the Jerusalem and Judea districts, potentially due to their distance from water, as Wang et al. [40] found that over 70% of drowning incidents among children under 5 occurred within 100 m of water. In Israel, access to swimming education is limited by geographic and socioeconomic factors, especially in rural areas. Addressing these disparities requires investments in infrastructure, subsidized programs, community outreach, equitable policies, and collaboration with nongovernmental organizations (NGOs) to ensure better water safety across all communities.

Analysis of the past 13 years indicates an increase in drowning incidents, especially from 2020 to 2022. This aligns with findings by Benihani et al., who reported a rise in drowning-related injuries in 2021 following the onset of the COVID-19 pandemic [22]. During the pandemic, increased home water exposure, reduced supervision, and gaps in water safety education likely raised drowning risks [41, 44, 45]. Additionally, climate change may have extended swimming seasons and intensified weather conditions, contributing to these risks [42, 43]. Addressing these trends requires a multifaceted approach, including public education, environmental safety measures, and targeted interventions for high-risk groups [46].

Trauma-related incidents, such as drowning, can cause severe, long-term neurological damage. A 2013 Helsinki study on 21 pediatric ICU drowning survivors found that at a median follow-up age of 12.5 years, 57% had neurological dysfunction, and 40% had intellectual impairments, with full-scale IQs below 80 [47]. These results underscore the need for early post-hospital assessment and long-term management of survivors, highlighting significant implications for healthcare systems. An integrated approach to preventing pediatric drowning is presented in Table 2.

**Table 2:** Prevention of pediatric drowning: an integrated approach

Prevention strategy	Description	Key studies
Public education and awareness	Increase awareness about drowning risks and preventive measures	[20, 46, 47]
Swimming skills acquisition	Encourage swimming lessons focusing on water safety and swimming proficiency	[21, 24]
Environmental safety measures	Implement pool fencing, safe water body design, and lifeguard services	[34, 48, 49]
Legislation and policy	Develop policies for pool safety, flotation device use, and water body access	[25, 48]
Emergency preparedness and response	Train in cardiopulmonary resuscitation PR and improve emergency medical services	[39, 48]
Targeted interventions for high-risk groups	Tailor programs for specific populations based on risk	[20, 46]
Research and surveillance	Conduct ongoing research and establish drowning registries	[35, 39]

Our study has limitations due to its retrospective design and multicenter approach, lacking detailed information on specific locations and activities, which limits targeted prevention recommendations. Variations in medical protocols across regions also affect data consistency. Despite these limitations, the national registry provides large-scale, population-wide data, enhancing generalizability and statistical power and allowing for longitudinal analysis of trends and health outcomes. While data from 2023 is not included, trends from 2010 to 2022 offer a solid foundation for adaptive drowning prevention strategies, allowing updates as new data emerge.

In conclusion, our study offers new insights into pediatric drowning by analyzing nationwide data over 13 years, revealing long-term trends and recent increases in incidents. We identified a bimodal age distribution with peaks in toddlers and teenagers, indicating the need for age-specific prevention strategies. Higher mortality rates in winter and spring highlight the importance of year-round safety campaigns, while regional disparities emphasize location-specific efforts. The link between prolonged hospitalization and higher mortality rates underscores the need for timely interventions. Our findings support a comprehensive prevention approach involving education, environmental safety, policies, and targeted strategies to enhance global drowning prevention efforts. Future research should focus on understanding the causes of recent increases in drowning incidents and developing targeted interventions to effectively mitigate these risks.

## Supplementary Information

Below is the link to the electronic supplementary material.

Supplementary file1 (DOCX 23.7 KB)

## Abbreviations

CBS: Central Bureau of Statistics
CPR: Cardiopulmonary resuscitation
ECLS: Extracorporeal life support
ED: Emergency department
ICD: International Classification of Diseases
ICU: Intensive care unit
MOH: Ministry of Health
NCDR: National Cause of Death Registry
NHDR: National Hospital Discharges Registry
NHEDR: National Hospital Emergency Department Registry
