# Supplementary material for: National trends in pediatric drowning — insights from the Israeli Ministry of Health registry-based cohort
Source: Eur J Pediatr. 2024 Sep 16;183(11):4921–8. doi: 10.1007/s00431-024-05771-5 (PMC11473486; doi:10.1007/s00431-024-05771-5)
Supplement: Supplementary file 1 — Supplementary file1 (DOCX 23.7 KB) [file 431_2024_5771_MOESM1_ESM.docx]

Supplemental Table 1. Demographics and characteristics of the identified cases who were treated in hospitals or died.

|  |  | **Drowned** | **Died on Scene** | **Died in ED** | **Died during hospitalization** | **Hospitalized and discharged** | **Discharged from ED** | **Died** | **% Hospitalized** | **95% CI** | **P-value** | **% Died** | **95% CI** | **P-value** | **Age adjusted rate** |
| --- | --- | --- | --- | --- | --- | --- | --- | --- | --- | --- | --- | --- | --- | --- | --- |
| Year^¥^ | 2010 | 141 | 3 | 5 | 3 | 91 | 39 | 11 | 68% | (59.6%, 75.8%) | 0.003 | 8% | ( 4.0%, 13.5%) | 0.059 | 5.6(4.7-6.6) |
|  | 2011 | 122 | 5 | 6 | 4 | 73 | 34 | 15 | 66% | (56.5%, 74.3%) | 0.003 | 12% | ( 7.0%, 19.5%) | 0.059 | 4.8(3.9-5.6) |
|  | 2012 | 161 | 4 | 6 | 6 | 81 | 64 | 16 | 55% | (47.3%, 63.3%) | 0.003 | 10% | ( 5.8%, 15.6%) | 0.059 | 6.2(5.2-7.1) |
|  | 2013 | 146 | 3 | 8 | 3 | 79 | 53 | 14 | 57% | (48.8%, 65.6%) | 0.003 | 10% | ( 5.3%, 15.6%) | 0.059 | 5.5(4.6-6.4) |
|  | 2014 | 125 | 6 | 5 | 8 | 64 | 42 | 19 | 61% | (51.1%, 69.3%) | 0.003 | 15% | ( 9.4%, 22.7%) | 0.059 | 4.6(3.8-5.4) |
|  | 2015 | 155 | 3 | 4 | 1 | 85 | 62 | 8 | 57% | (48.3%, 64.6%) | 0.003 | 5% | ( 2.3%, 9.9%) | 0.059 | 5.6(4.7-6.5) |
|  | 2016 | 158 | 5 | 5 | 7 | 81 | 60 | 17 | 58% | (49.3%, 65.5%) | 0.003 | 11% | ( 6.4%, 16.7%) | 0.059 | 5.6(4.7-6.6) |
|  | 2017 | 177 | 2 | 3 | 7 | 96 | 69 | 12 | 59% | (51.2%, 66.2%) | 0.003 | 7% | ( 3.6%, 11.5%) | 0.059 | 6.1(5.2-7.0) |
|  | 2018 | 182 | 1 | 9 | 17 | 79 | 76 | 27 | 53% | (45.5%, 60.5%) | 0.003 | 15% | (10.0%, 20.8%) | 0.059 | 6.2(5.3-7.1) |
|  | 2019 | 159 | 3 | 2 | 9 | 81 | 64 | 14 | 58% | (49.5%, 65.6%) | 0.003 | 9% | ( 4.9%, 14.3%) | 0.059 | 5.3(4.5-6.2) |
|  | 2020 | 150 | 1 | 2 | 6 | 83 | 58 | 9 | 60% | (51.4%, 67.7%) | 0.003 | 6% | ( 2.8%, 11.1%) | 0.059 | 5.0(4.2-5.8) |
|  | 2021 | 196 | 2 | 6 | 6 | 94 | 88 | 14 | 52% | (44.3%, 58.8%) | 0.003 | 7% | ( 4.0%, 11.7%) | 0.059 | 6.5(5.6-7.4) |
|  | 2022 | 229 |  | 3 | 10 | 111 | 105 | 13 | 53% | (46.2%, 59.4%) | 0.003 | 6% | ( 3.1%, 9.5%) | 0.059 | 7.5(6.6-8.5) |
| 2010-2022 |  |  |  |  |  |  |  |  |  |  |  |  |  |  |  |
| All | All | 2101 | 38 | 64 | 87 | 1098 | 814 | 189 | 57% | (55.3%, 59.6%) |  | 9% | ( 7.8%, 10.3%) |  | 5.7(5.5-6.0) |
| Sex^§^ | Female | 765 | 11 | 17 | 25 | 380 | 332 | 53 | 54% | (50.1%, 57.3%) | 0.010 | 7% | ( 5.2%, 9.0%) | 0.014 | 4.3(4.0-4.6) |
|  | Male | 1336 | 27 | 47 | 62 | 718 | 482 | 136 | 60% | (56.9%, 62.3%) | 0.010 | 10% | ( 8.6%, 11.9%) | 0.014 | 7.1(6.7-7.5) |
| District^†^ | Jerusalem | 262 | 9 | 12 | 17 | 139 | 85 | 38 | 62% | (55.4%, 67.7%) | 0.002 | 15% | (10.5%, 19.4%) | <0.001 | 4.6(4.0-5.2) |
|  | North | 380 | 7 | 3 | 13 | 173 | 184 | 23 | 50% | (44.7%, 55.1%) | 0.002 | 6% | ( 3.9%, 8.9%) | <0.001 | 6.6(5.9-7.2) |
|  | Haifa | 213 | 2 | 7 | 12 | 96 | 96 | 21 | 51% | (44.2%, 58.1%) | 0.002 | 10% | ( 6.2%, 14.7%) | <0.001 | 5.8(5.0-6.6) |
|  | Center | 498 | 7 | 14 | 7 | 276 | 194 | 28 | 58% | (53.1%, 62.1%) | 0.002 | 6% | ( 3.8%, 8.0%) | <0.001 | 5.9(5.4-6.5) |
|  | Tel-Aviv | 269 | 3 | 3 | 12 | 137 | 114 | 18 | 56% | (49.8%, 62.1%) | 0.002 | 7% | ( 4.0%, 10.4%) | <0.001 | 5.6(4.9-6.2) |
|  | South | 340 | 8 | 24 | 18 | 194 | 96 | 50 | 64% | (58.4%, 69.0%) | 0.002 | 15% | (11.1%, 18.9%) | <0.001 | 5.9(5.3-6.6) |
| Season | Winter | 152 | 6 | 7 | 6 | 68 | 65 | 19 | 51% | (42.3%, 59.0%) | 0.048 | 13% | ( 7.7%, 18.8%) | 0.002 |  |
|  | Spring | 434 | 8 | 23 | 25 | 219 | 159 | 56 | 57% | (52.4%, 62.0%) | 0.048 | 13% | ( 9.9%, 16.4%) | 0.002 |  |
|  | Summer | 1187 | 20 | 26 | 44 | 652 | 445 | 90 | 60% | (56.8%, 62.5%) | 0.048 | 8% | ( 6.1%, 9.2%) | 0.002 |  |
|  | Autumn | 328 | 4 | 8 | 12 | 159 | 145 | 24 | 53% | (47.2%, 58.3%) | 0.048 | 7% | ( 4.7%, 10.7%) | 0.002 |  |

Including unknown nationality or unknown district. Rates per 100,000.

^¥^Cochran-Armitage Trend Test; ^§^exact Fisher test; ^†^Pearson chi square

Supplemental Table 2. Adjusted drowning rate by year.

| Year | Adjusted rate | Negative | Positive |
| --- | --- | --- | --- |
| 2010 | 5.649802 | 0.932657 | 0.932657 |
| 2011 | 4.774407 | 0.847307 | 0.847307 |
| 2012 | 6.159995 | 0.951687 | 0.951687 |
| 2013 | 5.46743 | 0.886981 | 0.886981 |
| 2014 | 4.592603 | 0.805182 | 0.805182 |
| 2015 | 5.580242 | 0.87854 | 0.87854 |
| 2016 | 5.581219 | 0.870292 | 0.870292 |
| 2017 | 6.128159 | 0.90282 | 0.90282 |
| 2018 | 6.197811 | 0.900448 | 0.900448 |
| 2019 | 5.337136 | 0.829608 | 0.829608 |
| 2020 | 4.978016 | 0.796768 | 0.796768 |
| 2021 | 6.506207 | 0.91126 | 0.91126 |
| 2022 | 7.546273 | 0.978057 | 0.978057 |

Supplemental Table 3. Drowning rate by age.

| Age (years) | Lower CI | Rate | Upper CI |
| --- | --- | --- | --- |
| 0 | 6.037905 | 7.087258 | 8.266562 |
| 1 | 11.87913 | 13.33631 | 14.92291 |
| 2 | 11.8558 | 13.318 | 14.91075 |
| 3 | 9.791611 | 11.1314 | 12.60333 |
| 4 | 5.593095 | 6.623957 | 7.789765 |
| 5-9 | 2.863177 | 3.196328 | 3.557602 |
| 10-14 | 3.000941 | 3.358315 | 3.746537 |
| 15 | 4.467334 | 5.496561 | 6.691866 |
| 16 | 3.033814 | 3.899205 | 4.934693 |
| 17 | 5.457377 | 6.610174 | 7.934513 |
